# Phase 1 study of the pharmacokinetics and clinical proof-of-concept activity of a biofilm-disrupting human monoclonal antibody in patients with chronic prosthetic joint infection of the knee or hip

**DOI:** 10.1128/aac.00655-24

**Published:** 2024-07-16

**Authors:** Janet Conway, Ronald E. Delanois, Michael A. Mont, Alexandra Stavrakis, Edward McPherson, Edward Stolarski, Stephen Incavo, Daniel Oakes, Ralph Salvagno, John S. Adams, Adriane Kisch-Hancock, Edgar Tenorio, Anton Leighton, Stefan Ryser, Lawrence M. Kauvar, Nicholas M. Bernthal

**Affiliations:** 1 Rubin Institute for Advanced Orthopedics, Sinai Hospital of Baltimore, LifeBridge Health, Baltimore, Maryland; 2 University of California Los Angeles, Los Angeles, California, USA; 3 Gulfcoast Research Institute, Sarasota, Florida, USA; 4 Houston Methodist Hospital, Houston, Texas, USA; 5 University of Southern California, Los Angeles, California, USA; 6 Meritus Medical Center, Hagerstown, Maryland, USA; 7 Trellis Bioscience, Inc., Redwood City, California, USA; Providence Portland Medical Center, Portland, Oregon, USA

**Keywords:** biofilm, monoclonal antibody, prosthetic joint infection, antibiotic potentiation

## Abstract

We report the results of a first-in-human phase 1 clinical study to evaluate TRL1068, a native human monoclonal antibody that disrupts bacterial biofilms with broad-spectrum activity against both Gram-positive and Gram-negative species. The study population consisted of patients with chronic periprosthetic joint infections (PJIs) of the knee or hip, including both monomicrobial and polymicrobial infections, that are highly resistant to antibiotics due to biofilm formation. TRL1068 was administered via a single pre-surgical intravenous infusion in three sequentially ascending dose groups (6, 15, and 30 mg/kg). Concomitant perioperative antibiotics were pathogen-targeted as prescribed by the treating physician. In this double-blinded study, 4 patients were randomized to receive placebo and 11 patients to receive TRL1068 on day 1, as well as targeted antibiotics for 7 days prior to the scheduled removal of the infected implant and placement of an antibiotic-eluting spacer as the first stage of the standard of care two-stage exchange arthroplasty. No adverse events attributable to TRL1068 were reported. TRL1068 serum half-life was 15–18 days. At day 8, the concentration in synovial fluid was approximately 60% of the blood level and thus at least 15-fold above the threshold for biofilm-disrupting activity *in vitro*. Explanted prostheses were sonicated to release adherent bacteria for culture, with elimination of the implant bacteria observed in 3 of the 11 patients who received TRL1068, which compares favorably to prior PJI treatments. None of the patients who received TRL1068 had a relapse of the original infection by the end of the study (day 169).

**CLINICAL TRIALS:**

This study is registered with ClinicalTrials.gov as NCT04763759.

## INTRODUCTION

A substantial fraction of clinically meaningful bacterial infections are resistant to treatment due to the formation of biofilm, which shields bacteria from phagocytic cells and induces an antibiotic-refractory phenotype. By disrupting biofilm, TRL1068 allows access to biofilm-protected microbes and restores sensitivity to antibiotics, with potential benefits for many chronic infectious disease indications. Focusing on patients with a periprosthetic joint infection (PJI) of the knee or hip allowed direct documentation of biofilm reduction, employing an established analytical method. This initial study has provided evidence of efficacy, safety, as well as pharmacokinetic data to guide the design of future studies of TRL1068, in combination with conventional antibiotics, for chronic PJI and other biofilm-associated infections.

Bacteria within a biofilm are highly refractory to antibiotics, as their physiology shifts from a fast-growing planktonic state to a slower-growing sessile state (1). The biofilm also provides a shield against microbial phagocytosis. The full prevalence of bioﬁlms in human disease is likely underestimated as there are no widely accepted biomarkers. Nonetheless, biofilms are known to be present in a wide range of human tissues and are associated with a substantial fraction of all antibiotic-resistant disease conditions (2). Bacterial cell lysis and the resulting release of extracellular DNA (eDNA) contribute to the transformation into the biofilm state (3). Comparative genomic and proteomic studies have identified transcripts differentially regulated in the sessile state as compared to the planktonic state (4), including a family of bacterial proteins designated as DNA binding class II (DNABII, comprising the related IHF and HU protein families). Binding at random sites on the eDNA, which represents up to 50% of the biomass of the biofilm, DNABII proteins bend the linear eDNA into an acute angle that results in a multi-node scaffolding with a semi-rigid, three-dimensional web-like architecture (5). The importance of eDNA as a biofilm structural component was first established by showing that DNase is effective at disrupting biofilms *in vitro* (6), although the poor potency and high immunogenicity of DNase preclude using it as a pharmaceutical. Disruption of biofilm by polyclonal antibodies binding to DNABII proteins has also been described (7). TRL1068, described below, is the first monoclonal antibody developed against DNABII proteins.

### Chronic PJI overview

Lower extremity total joint arthroplasties are among the most commonly performed surgical procedures in the United States, with over 1.5 million surgeries per year (8–10). The projected number of annual cases of chronic PJI of the knee or hip in 2030 ranges from 50,000 to 125,000. Despite advances in perioperative antiseptic protocols, surgical technique, and operating room sterility, infection remains the most common etiology of prosthetic joint failure within the first year after primary arthroplasty (11). PJIs occur in 0.7%–2.2% of primary total hip arthroplasty or total knee arthroplasty (TKA) (12). The standard of care for chronic PJI is a two-stage exchange protocol consisting of removal of the infected implant, stabilization with an interpositional antibiotic-loaded spacer, followed by re-implantation of a new prosthesis after 6 weeks of systemic antibiotic treatment (13).

This two-stage revision protocol for chronic PJI had a 20% failure rate at 1 year in one major study (*n* = 470) (14). Even when successful, two-stage arthroplasty is highly morbid to patients as it involves two major surgeries, typically separated by 6–12 weeks, including prolonged treatment with local and systemic antibiotics. Stage 1 typically encompasses the removal of the infected prosthesis, an extensive surgical debridement, and the insertion of a spacer secured by cement that elutes antibiotics locally as part of an aggressive antibiotic regimen aimed at sterilizing the joint site prior to the replacement of the spacer with a new prosthetic joint. Although chronic PJI may result in a loose prosthesis, there are major challenges for the surgeon in removing a well-fixed prosthesis, notably the complete removal of the cement, which poses a risk of severe damage to the remaining bone stock (15). Two-stage protocols impart a substantial medical and economic burden as patients are rendered immobile, with prolonged hospitalizations and extended use of parenteral and oral antibiotics.

### TRL1068 overview

As previously published (16, 17), the gene for TRL1068 was cloned from human memory B lymphocytes. Its protein product binds with high affinity (Kd ~50 pM) to an epitope on DNABII proteins conserved across nearly all clinically relevant Gram-negative and Gram-positive bacteria, for which no homologs in the human genome were identified by sequence analysis.

The high affinity of TRL168 is important to achieve permanent removal of the DNABII protein, which might otherwise provide building blocks for biofilm formation. A direct demonstration of broad-spectrum efficacy was shown using the established 96-well microplate minimum biofilm eradication concentration assay, also called the Calgary Biofilm Device (Innovotech, Edmonton, Canada). Efficacy studies of TRL1068 in two established murine models of infected implants have demonstrated the qualitative potentiation of conventional bactericidal antibiotics (16, 17). In both models, antibiotic treatment for a *Staphylococcus aureus* biofilm infection was combined with TRL1068 administered at 15 mg/kg; compared to antibiotics alone, animals receiving TRL1068 had a 2–3 log reduction in colony-forming units (CFUs) within 6 days. Analogous efficacy has been published for a representative Gram-negative pathogen, *Acinetobacter baumannii*, in a mouse model of skin and soft tissue catheter-related infection (18); similar results have also been observed in unpublished studies using *Pseudomonas aeruginosa*. Furthermore, biofilm disruption achieved *in vivo* by TRL1068 alone (without antibiotics) was visualized *ex vivo* by scanning electron microscopy; the three-dimensional biofilm matrix was abolished on the implant (16).

To assess prophylactic activity, TRL1068 was administered 24 h prior to infection in an infected plastic cage mouse model, resulting in no detectable infection on day 6 (17). Similarly, an extended follow-up period after treatment in a rodent model for infected metal prostheses (14 days after cessation of all treatment) provided time for recurrence of the biofilm-protected infection. No recurrence was detectable in 26 out of 27 treated animals, with the remaining one showing a 2-log reduction in CFUs compared to antibiotics alone (16).

The gene for TRL1068 was cloned from a healthy human blood donor using a proprietary single-cell detection method called CellSpot (Trellis Bioscience, Redwood City, CA, USA), suggesting this monoclonal antibody would be safe for humans. Antibodies against the DNABII family of bacterial proteins were detected in the memory B-cell compartment of all 20 healthy human donors surveyed during the discovery program (19), indicative of prior humoral responses to DNABII proteins. However, circulating antibodies were not detectable by enzyme-linked immunosorbent assay (ELISA) in the serum of these healthy blood donors. Likewise, each participant in the present phase 1 trial was evaluated for baseline levels of DNABII antibodies prior to dosing, with none found in these chronic PJI patients.

In a rat toxicology model for evaluation of TRL1068, there were no clinical observations or findings in the assessment of functional operational battery, respiratory function, body weight, ophthalmology, hematology, coagulation, clinical chemistry, urinalysis, organ weight, or macroscopic and microscopic analyses that could be attributed to the twice-weekly intravenous administration of TRL1068 over a period of 28 days at doses of up to 196 mg/kg/dose.

Here, we present the results of a phase 1 clinical study (NCT04763759) evaluating the pharmacokinetics (PK), tolerability, and preliminary efficacy of TRL1068 administered as a single intravenous 60-minute infusion (three doses evaluated in total across the three dose groups). This first-in-human study of TRL1068 was conducted in volunteers who had a chronic bacterial periprosthetic joint infection of the knee or hip. Recruitment of chronic PJI patients instead of healthy volunteers facilitated assessment of TRL1068 penetration into the infected synovial fluid at 7 days after TRL1068 infusion, allowing comparison to serum PK levels as well as exploratory pharmacodynamics evaluation within the limited time window of treatment. TRL1068 was studied as a prelude to the standard of care for two-stage arthroplasty. TRL1068 was administered on day 1, followed by daily antibiotic administration. On day 8, patients began the two-stage arthroplasty, which included the removal of the infected implant and the placement of an interpositional spacer, followed by a 6-week course of parenteral antibiotics. At that time, patients were eligible for re-implantation of a new prosthesis, pending a negative aspiration culture and normalized synovial biomarkers of inflammation. Outcomes evaluated included PK, safety, implant-associated bacterial pathogen CFUs following removal of the infected implant, and infection recurrence by day 169 (end of study).

## MATERIALS AND METHODS

### TRL1068

TRL1068 is a human IgG1 kappa [G1m1,17 (z,a); Km3 allotype] monoclonal antibody with a calculated isoelectric point of 9.23. The bulk drug product was manufactured by AGC Biologics, Inc. (Bothell, Washington, USA) using good manufacturing practices in Chinese hamster ovary cells.

### Bioanalytical methods for PK analysis

Quantitative determination of TRL1068 in serum and synovial fluid samples was conducted using a validated ELISA method by Immunologix, Inc. (Tampa, FL, USA). A biotinylated epitope peptide was immobilized on a streptavidin-coated polystyrene microplate. Captured TRL1068 was detected using an affinity-purified goat anti-human kappa light-chain peroxidase conjugate antibody (Sigma-Aldrich Corp., St. Louis, MO, USA). Following incubation with a peroxidase substrate (tetramethylbenzidine), optical density readings were used to calculate TRL1068 concentrations from a four-parameter logistic regression curve fit to reference standards using SoftMax Pro v.5.4.5 (Molecular Devices, Inc., Sunnyvale, CA, USA).

A serum assay of immunogenicity [anti-drug antibodies (ADAs)] was conducted using a validated electrochemiluminescence bridging assay from Meso Scale Discovery, Inc. (Rockville, MD, USA) by Immunologix, Inc.; biotinylated TRL1068 was immobilized on a streptavidin-coated gold-surfaced microplate. Captured ADAs were detected with TRL1068 labeled with a SULFO-TAG following 90 minutes of incubation at room temperature. A Meso QuickPlex SQ 120 reader measured the intensity of relative light units, which was proportional to the amount of anti-TRL1068 antibody present.

### Study design and participants

**Fig 1:**
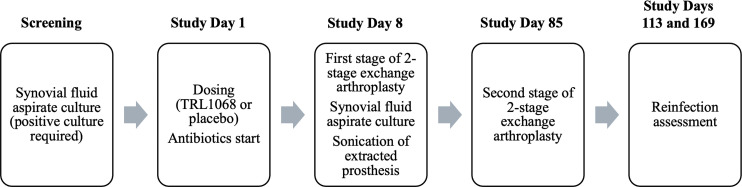
Study design overview. Timeline is shown for a double-blinded, placebo-controlled study of patients with chronic prosthetic joint infection of the knee or hip.

The study, which was double blinded and placebo controlled, enrolled 15 chronic PJI patients presenting with inflammation more than 30 days after implantation and confirmed as a bacterial infection by synovial fluid aspirate culture; all patients had well-fixed prostheses. Of these, 11 were treated with a single 60-minute infusion of TRL1068 in addition to microbe-targeted antibiotics, while four received a placebo (saline, indistinguishable from the TRL1068 infusion) in addition to microbe-targeted antibiotics, whose selection was based on baseline synovial fluid aspirate cultures. Patients who met all entry criteria at screening were randomized on day 1 to receive TRL1068 or placebo, utilizing a central randomization system. Inclusion criteria included 18–85 years of age and a body mass index of <40. Exclusion criteria included evidence of active infection other than bacterial PJI of the knee or hip, a Child-Pugh score of >6 to exclude moderate to severe liver disease, congestive heart failure, chronic obstructive pulmonary disease, an immunocompromised state, malignancy, autoimmune disease, uncontrolled diabetes (hemoglobin A1C >7.0), and clinically meaningful electrocardiogram or clinical lab abnormalities. These inclusion and exclusion criteria ensured that the population with a localized infection was otherwise reasonably healthy so as not to confound safety findings due to significant underlying comorbidities. The study design is summarized in Fig. 1.

The exchange arthroplasty timelines in Fig. 1 were defined at the outset of the study. However, patient management after explantation, including duration and type of antibiotic treatment, was at the discretion of each investigational site. Because the molecular weight of TRL1068 is greater than 100,000 Da and the drug is administered intravenously, interspecies comparisons of doses were appropriately made on a milligram per kilogram basis rather than on the basis of doses normalized for total body surface area: dose level 1 = 6 mg/kg (three experimental and one placebo); dose level 2 = 15 mg/kg (five experimental and two placebo); dose level 3 = 30 mg/kg (three experimental and one placebo). With no toxicity detected in the pre-clinical toxicology studies, the no-observed-effect level in rats was determined to be 196 mg/kg; the starting dose in humans thus had a >30-fold safety margin. An independent study monitoring committee reviewed unblinded safety, dose escalation, and overall timelines, including adverse events potentially related to TRL1068.

### Observational correlation of efficacy

Sonication of freshly explanted prosthetic knee or hip joints was introduced in 2007 as a tool for identifying the bacterial species to facilitate targeted antibiotic treatment via the addition of antibiotics to the cement used for the fixation of the spacer. The original publication in the field by Trampuz et al. (20) at the Mayo Clinic (Rochester, MN, USA) included 79 subjects. However, that number included 2 with fungal infections (an exclusion criterion in the TRL1068 trial) and a further 17 with aseptic PJI, which was also an exclusion criterion for the TRL1068 study; the remaining 60 subjects were used as historical controls for comparison to the TRL1068 trial results presented here.

The standardized method, conducted at each of the clinical sites, provides sonication in 400-mL Ringer’s solution for 5 minutes at 40 ± 2 kHz and a power density of 0.22 ± 0.04 W/cm^2^ in an Aquasonic Model 750T ultrasound bath (VWR Scientific Products, Radnor, PA, USA). This protocol has been utilized in multiple publications since then (8, 21). In addition to clarifying what bacterial species were present, the initial publication provided a standardized procedure for a quantitative assessment of the adherent, biofilm-associated bacterial burden, with the resultant data normalized to CFU per milliliter of the unconcentrated sonication fluid. The CFUs are based on counting colonies on a Petri plate, with a limit of detection of 2 CFUs and a practical limit of 100 CFUs before colony overlap made quantitation unreliable. Accordingly, at or above 100 CFUs, as used in the Trampuz et al. study, was used in the present analysis as a threshold value for comparing populations.

### Study design and patient demographics

The focus on patients who had a PJI enabled collection of safety and PK data for TRL1068 in combination with antibiotics. There were five sites that enrolled subjects. The baseline characteristics of enrolled PJI patients are provided in Table 1. See Table S1 for additional details regarding subject histories.

**TABLE 1:** Baseline characteristics of patients with prosthetic joint infection^*a*^

Parameter	TRL1068 (*n* = 11)	Placebo (*n* = 4)
Age (years), median (range)	65 (52–80)	65 (38–84)
Sex at birth, *n* (%)		
Female	6 (55)	0 (0)
Male	5 (45)	4 (100)
Race, *n* (%)		
White	10 (90)	3 (75)
Black or African American	1 (9)	1 (25)
Median BMI (range)	31.00 (20.5–38.3)	23.40 (20.4–32.0)
Weight (kg), median (range)	87.10 (54.3–110.7)	84.15 (70.3–98.2)

^*a*^ BMI, body mass index.

## RESULTS

### PK parameters

Consistent with expectations for an infused human IgG1 antibody, the mean serum half-life of TRL1068 administered intravenously was 20 days, with little variability in *C*_max_ and area under the curve (AUC). The sample size for PK assessments was 11 subjects (*n* = 3 at 6 mg/kg, *n* = 5 at 15 mg/kg, and *n* = 3 at 30 mg/kg). Increases in the AUC with dosage were consistent across all three levels. The systemic half-life range was 15–18 days (Table 2).

**TABLE 2:** Serum pharmacokinetics analysis of TRL1068

	Cohort 1 (6 mg/kg) *n* = 3	Cohort 2 (15 mg/kg) *n* = 5	Cohort 3 (30 mg/kg) *n* = 3
Mean *C*_max_ (µg/mL)	150 ± 30	398 ± 43	784 ± 105
Half-life (days)	18.5 ± 8.5	15.1 ± 6.9	15.1 ± 4.1
*T*_max_ (h)	3.0 ± 2.7	4.2 ± 4.9	1.7 ± 0.6
AUC_0-last_ (μg *× n* = h/mL)	22,170 ± 1,826	53,392 ± 8,565	109,717 ± 21,314

To determine the bioavailability of TRL1068 in synovial fluid, a needle aspirate of the infected joint was performed on day 8 (approximately 7 days after the single-dose infusion) immediately prior to the removal of the infected prosthesis (Fig. 1). The mean synovial fluid concentration was proportional to the dose: 21.1 ± 9.1 µg/mL at 6 mg/kg, 57.3 ± 20.3 µg/mL at 15 mg/kg, and 134.3 ± 12.0 µg/mL at 30 mg/kg. On average, the synovial fluid aspirate had approximately 60% of the same subject’s serum concentration (Table 3). In pre-clinical studies, the effective concentration for TRL1068 was 1.5 µg/mL. Dissolution of the biofilm requires the removal of multiple copies of the DNABII proteins from the biofilm matrix, leading to a very steep dose-response curve (high cooperativity); accordingly, this concentration represents a threshold for efficacy.

**TABLE 3:** Concentrations of TRL1068 in serum and synovial fluid on day 8^*a*^

	Cohort 1 (6 mg/kg) *n* = 3	Cohort 2 (15 mg/kg) *n* = 4	Cohort 3 (30 mg/kg) *n* = 3
Mean concentration in blood serum (µg/mL)	32.3 ± 4.0	102.4 ± 26.6	232 ± 54.0
Mean concentration in synovial fluid (µg/mL)	21.1 ± 9.1	57.3 ± 20.3	134.3 ± 12.0
Mean % of blood serum in synovial fluid (%)	65.4	59.8	60.4

^*a*^ No synovial fluid for PK analysis was taken from patient 5 in cohort two on day 8.

### Safety

TRL1068 was well tolerated. No signs of dose-limiting tolerability or infusion reactions were observed. No drug-related adverse events were reported by the investigators or determined by the study monitoring Committee. See Table S2 for details.

Immunogenicity, measured as circulating ADAs, was evaluated for all patients who received TRL1068. There were two patients who presented with a positive ADA result at enrollment; however, these baseline levels were unchanged at the pre-defined post-dose assessment timepoints. All other patients had no value above the detection threshold, either at baseline or throughout the study.

### Quantitation of CFUs

Assessment of TRL1068 efficacy was achieved by determining the CFUs of any bacterial pathogens released from the explanted prosthesis by sonication. In the TRL1068 PJI population, all patients had a pre-operative synovial fluid aspirate yielding a positive culture (a key inclusion criterion for the study) that strongly suggests the presence of biofilm on the prostheses. Since the placebo group was small (*n* = 4), we compared these results to those of an untreated historical control group at the Mayo Clinic reported by Trampuz et al. in the study that established the sonication protocol used here (20). All patients in the Trampuz et al. study had a diagnosis of chronic PJI, but not all were verified as culture positive in synovial fluid samples taken prior to explanting the infected prosthesis. Patients with an unverified culture status were excluded from the analysis, leaving the historical control group at 60 patients with a similar distribution of pathogens as in the present study.

Using the Trampuz et al. study threshold value of 100 CFUs in the sonication fluid as an indicator of a substantial surface-associated bacterial burden, 51 out of 60 (85%) of the explanted hip or knee prostheses met or exceeded that value. For the TRL1068 population (*n* = 11), only four were at or above that level (36%); three of those involved staphylococcal species (Table 4). Moreover, three of the patients treated with TRL1068 had undetectable CFUs in the sonication solution. There were four patients who had polymicrobial infections, and at least one of the species present in the pre-treatment synovial fluid sample for these patients had been eliminated by the time the infected prosthesis was removed 7 days after treatment with TRL1068. TRL1068 treatment eradicated a diverse set of bacterial species (number of examples in parentheses): *Streptococcus* (3), *Pseudomonas* (2), *Klebsiella* (1), and *Enterobacter* (1). For *Staphylococcus* spp. (5), two had <100 CFUs in the sonication fluid.

**TABLE 4:** Subjects and infection status^*a*^^,^^*b*^

Patient ID	Dose level (mg/kg)	Baseline pathogens in synovial fluid	Culture of sonication fluid of extracted prosthesis	Sonication fluid of prosthesis (CFU/mL)
1	6	*Staphylococcus lugdunensis*	*S. lugdunensis*	13
2	6	*Cutibacterium acnes*	*Cutibacterium acnes*	10
4	6	*Pseudomonas aeruginosa*	*P. aeruginosa*	1
5	15	*Enterobacter cloacae* *Stenotrophomonas maltophilia*	No growth	0
			*Stenotrophomonas maltophilia*	33
			*Oligella urethralis*	50
			*Actinomyces* sp.	50
			*Corynebacterium* sp.	>100
			*Prevotella melaninogenica*	>100
			*Campylobacter ureolyticus*	>100
7	15	CoNS	CoNS	50
8	15	CoNS	*S. lugdunensis*	>100
10	15	*Streptococcus pneumoniae*	No growth	0
		*Enterococcus faecalis*	*E. faecalis*	15
11	15	*Streptococcus agalactiae*	No growth	0
12	30	*Pseudomonas aeruginosa*	No growth	0
		*Klebsiella* sp.	No growth	0
13	30	*Streptococcus mitis* group	No growth	0
		*S. lugdunensis*	*S. lugdunensis*	>100
15	30	*S. lugdunensis*	*S. lugdunensis*	>100
3	Placebo	*S. epidermis*	CoNS	20
6	Placebo	*P. aeruginosa*	*P. aeruginosa*	100
9	Placebo	No synovial fluid	na	na
14	Placebo	*Corynebacterium*	*Corynebacterium*	>100

^*a*^ CFU, colony-forming unit; CoNS, coagulase-negative *Staphylococcus*; na, not available.

^*b*^ Patient 5 had a full thickness wound dehiscence, including the joint capsule, exposing the total knee prosthesis.

There was considerable variability in the severity of PJI in our patient population, including the presence and degree of draining sinuses and tissue defects. Patient 5 was an extreme example. This 66-year-old woman presented with an infected total knee prosthesis with extensive wound dehiscence, a complex three-dimensional contour defect, and an extensor mechanism deficiency. Parts of the prosthesis were clearly visible through the dehiscence. Baseline culture of a synovial aspirate on day 1 identified two pathogens, *Enterobacter cloacae* and *Stenotrophomonas maltophilia*, that were no longer present in the pre-operative synovial fluid aspirate on day 8. However, *Stenotrophomonas maltophilia* and several additional bacterial pathogens not detected in the baseline synovial fluid aspirate were identified following culture of the sonication fluid of the explanted prosthesis (Table 4).

### Recurrence

All patients received standard care, including antibiotics, subsequent to the explantation of the prosthesis on day 8. There were none of the 11 patients treated with TRL1068 who experienced a relapse infection by the same bacterial species at the end of the 6-month observation period, including patient 5 (Table 4). There was one of the four placebo controls who had a relapse (*Pseudomonas*).

## DISCUSSION

Biofilm is a major contributor to antibiotic treatment failures in a variety of clinical settings. Biofilm disruption thus represents an important clinical treatment strategy that is being pursued by a variety of approaches, including quorum-sensing inhibitors, metal oxide nanoparticles, anti-microbial peptides, bactericidal phages or phage-derived proteins, and intensive antibiotic exposure (22). Meanwhile, the number of implanted medical devices is steadily increasing (23).

In chronic PJI, extensive clinical experience has established that antibiotics alone do not eliminate the infection, with persistence generally attributed to biofilm formation (24). This is why the standard of care for chronic PJI in the United States is to remove the infected prosthesis. Moreover, antibiotic treatment at subtherapeutic dosing levels can select for specific bacterial resistance mutations, compounding the resistance due to biofilm (25). There are currently approximately 1.5 million total knee and hip arthroplasties performed annually in the United States (9), which is projected to exceed 2 million by 2030 (10, 26). Despite advances in surgical technique, PJI occurs in 0.7%–2.2% of primary procedures (12), with a 20% rate of relapse infections at 1 year for chronic PJI patients (14). The recurrent infection in one of the four placebo patients in the present study is consistent with this historical rate.

The TRL1068 antibody’s 11-residue-binding epitope is found in hundreds of both Gram-positive and Gram-negative bacterial species, including all of the World Health Organization’s list of priority ESKAPE pathogens (*Enterococcus faecium*, *Staphylococcus aureus*, *Klebsiella pneumoniae*, *Acinetobacter baumannii*, *Pseudomonas aeruginosa*, *and Enterobacter spp*.) (17). This breadth of binding to homologs of the target protein across extensive phylogenetic distances is of particular value for the treatment of polymicrobial infections, which represent up to a third of PJI infections (27, 28).

For staphylococcal infections, which are the most common cause of chronic PJI, the phase 1 clinical data indicated efficacy trends, although not as extensive as for most other bacterial species within the 7-day time frame of assessment of anti-microbial activity prior to the scheduled explant. A potential explanation for this finding is that the staphylococcal biofilms have fewer eDNA-associated DNABII-binding proteins than the biofilms of most other bacterial species (29), relying instead on a greater level of aggregated proteins surrounding the bacteria, including fibrinogen and albumin (30). Furthermore, the interval between TRL1068 treatment and prosthesis removal was brief. The 7-day exposure period was selected as sufficient for assessment of safety, tolerability, and PK while not unduly prolonging the time to provide patients with a standard of care for a two-stage procedure. Pre-clinical mouse data showed that full penetration of TRL1068 into a subcutaneously placed hollow perforated Teflon tube was not achieved until at least 3 days (17). The distribution of the antibody and the antibiotic into the bone/cement-implant interface in humans may be even slower. In addition, some of the antibiotics given (e.g., tetracycline) are not bactericidal and/or may take days to achieve maximal effective levels within the extravascular area of infection.

All of the patients enrolled in the study had chronic PJI, which may have developed and expanded over months, as exemplified by patient 5. This patient had previously had a two-stage revision of a PJI and presented in our study with extensive tissue defects; the resulting open wound exposed a part of the prosthesis surface to further colonization by bacterial pathogens from the skin or other sources. Due to a lack of blood flow in the exposed area, systemic TRL1068 and antibiotic therapy would have had limited opportunity to counter such new infections. We propose that the pathogens identified through sonication of patient 5’s extracted prosthesis on day 8 were on the exposed surface of the prosthesis that was not accessible to blood-borne TRL1068. For the area that was covered with tissue and thus fully exposed to TRL1068, the *Enterobacter* biofilm was completely eliminated. Upon removal of the infected prosthesis, the complex tissue defects were reconstructed, and parenteral antibiotics were administered based on the standard of care for the full range of identified pathogens. This patient had no recurrent PJI at study end.

Clinically relevant resistance to TRL1068 is expected to be rare. Mutations in the bacterial epitope region are known to alter DNA binding (31), suggesting that TRL1068 escape mutations could decrease the functionality of this essential site on the protein. Furthermore, DNABII proteins are dimers, including heterodimers, implying that escape will often require concurrent mutations in more than one gene. Also, the DNABII protein is released into the extracellular matrix following bacterial cell lysis, meaning that an escape mutant would have no growth advantage as it would already be dead by the time that its DNABII proteins were exposed to TRL1068. In addition, we did not observe clinically relevant levels of circulating antibodies against TRL1068. This is important since the presence of such anti-drug antibodies for other antibody therapeutics has been shown to affect the PK or efficacy (32).

### Conclusions

The first-in-human results described here are consistent with pre-clinical efficacy data and support further development of TRL1068 for the treatment of biofilm-associated infections, including medical device-associated infections and bacteremia. The phase 1 study has established that TRL1068 attains effective extravascular tissue concentrations within 7 days of TRL1068 infusion, as measured in synovial fluid. The mean systemic half-life of 20 days and lack of neutralizing antibodies suggest the possibility of long-term treatment with repeated dosing to address a wide variety of biofilm-associated infections. No drug-related adverse events were observed. The reduction in periprosthetic joint implant-associated bacterial infection was compared favorably to historical controls, with efficacy trends observed across a diverse set of bacterial species. None of the TRL1068-treated patients had a recurrence of the original infection; if confirmed in a larger phase 2 study, this result will represent a substantive advance in the management of PJI. Taken together, the results of this initial exploratory study have provided evidence of efficacy, safety, and pharmacokinetic data to guide the design of phase 2 and 3 studies of TRL1068, in conjunction with targeted antibiotic therapy, for addressing a wide variety of biofilm-associated infections.

## SUPPLEMENTAL MATERIAL

Supplemental tables aac.00655-24-s0001.pdf Tables S1 and S2.

ASM does not own the copyrights to Supplemental Material that may be linked to, or accessed through, an article. The authors have granted ASM a non-exclusive, world-wide license to publish the Supplemental Material files. Please contact the corresponding author directly for reuse.
