# Supplementary material for: Phase 1 study of the pharmacokinetics and clinical proof-of-concept activity of a biofilm-disrupting human monoclonal antibody in patients with chronic prosthetic joint infection of the knee or hip
Source: Antimicrob Agents Chemother. 2024 Jul 16;68(8):e00655-24. doi: 10.1128/aac.00655-24 (PMC11304715; doi:10.1128/aac.00655-24)
Supplement: Supplemental tables — Tables S1 and S2. [file aac.00655-24-s0001.pdf]

Supplementary Table S1: Subject Demographics, PJI History, & Outcomes (Treated and Placebo)

Patient ID	Dose Level (mg/kg)	Age & Gender	Comorbidities	PJI Location & History	Sinus Present?	Antibiotics & Duration	Reinfection or New Infection?	Further Surgery w/in 6 Months?	Survival at 6 Months?
1	6	55yo M	Aortic ectasia thoracic, osteoarthritis (bilaterial knees), hyperlipidemia, hypertension, vitamin B12 deficiency (non-anemic), allergic rhinitis, seasonal allergies, fatty liver, sebaceous cyst, sciatica, tobacco use, vitamin D deficiency, toe fungus (left and right big toe), anemia (only after surgeries)	R knee 6 months prior to enrollment	Not at study entry, develope d 4 months into study	Doxycycline ongoing	New infection with rare *Staphylococcus aureus*	No	Yes
2	6	72yo M	Osteoarthritis, sleep apnea, hyperlipidemia, hiatal hernia, nonobstructive coronary artery disease, BPH, atrial fibrillation, rosacea, GERD	R knee 6 months prior to enrollment	No	Doxycycline Monohydrate D1-D9 Augmentin D9-10	No	No	Yes
4	6	54yo F	Smoker, knee pain bilateral, osteoarthritis, sleep apnea, constipation, hyperlipidemia	L knee Unknown	No	Ciprofloxacin D1-D49	No	No	Yes
5	15	66yo F	Osteoarthritis, depression, hyperlipidemia, slipped disk, hypertension	L knee 3 months prior to enrollment	Yes	Bactrim D1-D7 Cefepime & Cefazolin D7 Vancomycin D11-D21 Levofloxacin D10-D20	No	No	Yes

7	15	67yo M	Prostate enlargement, osteoarthritis, hypertension	R knee 3-4 months prior to enrollment	No	Doxycycline D1-D8 Vancomycin D8-D158	No	No	Yes
8	15	80yo M	Thoracolumbar scoliosis idiopathic and degenerative, hyperlipidemia, rotator cuff arthropathy, chronic draining sinuses (right hip, two posterior and one lateral), left shoulder arthritis with impingement, right shoulder arthritis, pre-diabetic, hip arthritis (left), macular degeneration, hypertension, bilateral knee arthritis (moderate right, mild left), nontraumatic tear of supraspinatus tendon (right)	R hip 9-10 years prior to enrollment	Yes	Doxycycline 10 years prior to study entry through D10 Cefazolin D7-D9 and D133-134 Clindamycin D14-D28	No	Yes, second stage completed	Yes
10	15	66yo F	Hiatal hernia, back pain	R hip 2 months prior to enrollment	Yes	Augmentin D1-D8 Unasyn (Ampicillin/Sulbactum) D12-D52	New infection with *Candida albicans*	Yes, second stage completed	Yes
11	15	52yo F	Hypertension (diet controlled), regional chronic pain syndrome, anxiety, depression, fibromyalgia, bilateral foot Raynard's syndrome, cefepime	R hip 5 years and 4 months prior to enrollment	No	Doxycycline 4 years prior to study entry-D1 Cefazolin D10-D114 Daptomycin D116-D118 Vancomycin D116 Daptomycin D118-study end	New infections *Enterobacter cloacae complex*, *Bergeyella zoohelcum*, and	Yes, two antibiotic spacer exchanges	Yes

			allergy (causes rash), latex allergy (causes rash)				*corynebacterium species*		
12	30	68yo M	Hypertension, hyperlipidemia, osteoarthritis	L hip 1 year and 2 months prior to enrollment	Yes	Cefepime D9-study end	No	No	Yes
13	30	75yo F	Hypertension, hyperlipidemia	L knee 2 weeks prior to enrollment	No	Minocycline D1-D7<br>Amoxycillin D1-D7<br>Vancomycin D10-D51<br>Cefazolin D97<br>Cephalexin D97-D98<br>Minocycline D98-D128	No	Yes, second stage completed	Yes
15	30	62yo F	Amnesia, menopause, osteoarthritis of cervical spine, FOM (frequency of micturition), vitamin B12 deficiency, primary insomnia, sensorineural hearing loss (SNHL) of both ears, hot flashes, atrophic vaginitis, iron deficiency anemia, essential hypertension, atrial premature contractions, constipation	L knee 2 years 2.5 months prior to enrollment	No	Cefadroxil D1-D8<br>Vancomycin D8-D41<br>Cefazolin D9-D40	No	No	Yes
3	Placebo	66yo M	Allergy to eggs, osteoarthritis	R knee 2-12 months prior to enrollment	No	Doxycycline Monohydrate D1-D8<br>Cefazolin D12-D48	No	No	Yes

				previous revision 3 years prior to enrollment					
6	Placebo	38yo M	Deep venous thrombosis of the left upper extremity unclear chronicity, insomnia (due to pain), itching due to pain medication, post-traumatic arthritis of right knee, thrombocytosis, chronic pain (due to right knee injury from motorcycle accident), hardware associated chronic osteomyelitis of right femur with draining sinus, acute blood loss anemia after surgery, desiccated cervical spine disc, daptomycin allergy with fevers and rash	R knee 1 week prior to enrollment	Yes	ceftolozane-tazobactam 2 weeks prior to study entry through D17 levofloxacin D16-study end	Reinfection with *Pseudomonas aeruginosa*	No	Yes
9	Placebo	71yo M	BPH	R hip 3-3.5 years before enrollment	No	Linezolid D1-D58	No	No	Yes
14	Placebo	84yo M	N/A	L knee 2.5 months prior to enrollment	No	Linezolid D1-D9 Vancomycin D9-study end	No	No	Yes

Supplementary Table S2: Adverse Events (Treated and Placebo)

Patient ID	Dose Level (mg/kg)	Event Title(s)	Serious?	Day of Onset	Day of Resolution	Relation to IP
1	6	Hiccups	No	D10	D17	Unlikely
		Post-operative acute blood loss anemia	No	D10	D101	Unlikely
		Recurrent wound draining sinus	Yes	D29 & D48	D29 & D48	Unlikely/remote
2	6	Bilateral ankle swelling and erythema resembling cellulitis	Yes	D19	D19	Unrelated/not related
8	15	Paresthesia of tongue and throat itchiness	No	D1	D1	Unlikely, infusion reaction[1]
		Left wrist and left shoulder muscle sprain due to fall	No	D121	D121	Unlikely
10	15	Right hip total dislocation resulting in revision surgery	Yes	D142	D142	Unlikely/remote
11	15	Anemia postoperative antibiotic spacer exchange	Yes	D115	D115	Unrelated/not related
		Right hip wound dehiscence with serosanguinous drainage resulting in right hip antibiotic spacer exchange with debridement and washout	Yes	D147	D147	Unrelated/not related
12	30	Worsening anemia	No	D66	D66	Unlikely
15	30	Nausea	No	D2	D2	Unlikely, not infusion related, site thinks likely due to antibiotics
15	30	Intermittent nausea	No	D8	D18	Unlikely, site thinks likely due to antibiotics
15	30	Constipation post-op	Yes	D8	D20	Unlikely
15	30	Vomiting	No	D20	D20	Unlikely
15	30	Rash, termed DRESS syndrome	No	D37	D53	Unlikely, site thinks may be due to antibiotics
15	30	High blood pressure	No	D44	N/A, ongoing	Unlikely

6	Placebo	Intermittent generalized itching	No	D4	D102	Unlikely
		Wound drainage	Yes	D43	D121	Unrelated/not related

[1] Mild, deemed unlikely related to study drug by the PI and Study Monitoring Committee (SMC). Administration of diphenhydramine (Benadryl®IV) can lead to drowsiness and therefore could have positively affected this subject's presumed anxiety during study drug infusion; subject may have been excited and intermittently hyperventilating, which was ameliorated by the sedative effects of Benadryl®IV. If it were an allergic reaction, it is unlikely that it would resolve so quickly and completely. No signs of Type 1 reaction (flushing, pruritus, urticaria, shortness of breath, hypotension, and life-threatening anaphylaxis) were observed.

- Timeline:
  - 0955 Infusion start
  - 1100 Tingling tip of tongue, infusion paused
  - 1107 12.5 mg Benadryl®IV
  - 1119 Infusion restarted
  - 1122 Infusion completed
  - 1200 Resolution of tingling of tongue
  - 1222 Throat itchiness
  - 1235 12.5 mg Benadryl®IV
  - 1322 Resolution of throat itchiness
